# Supplementary material for: Differential metabolism between biofilm and suspended Pseudomonas aeruginosa cultures in bovine synovial fluid by 2D NMR-based metabolomics
Source: Sci Rep. 2022 Oct 15;12:17317. doi: 10.1038/s41598-022-22127-x (PMC9569359; doi:10.1038/s41598-022-22127-x)
Supplement: Supplementary file 1 — Supplementary Information. [file 41598_2022_22127_MOESM1_ESM.pdf]

Supplementary Figures

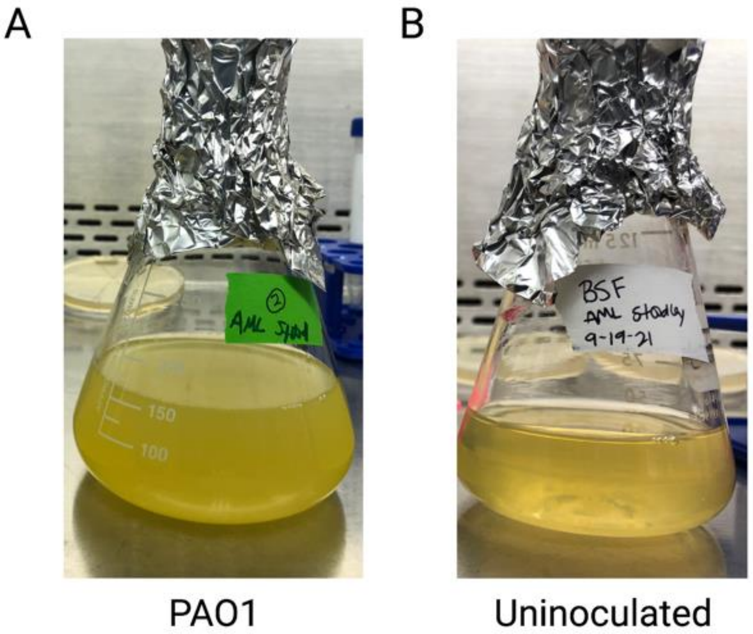

S1 Fig. Photos of shaking cultures after 24 hrs of incubation show the 50% BSF/PBS culture inoculated with PAO1 (A) and uninoculated as a control (B). The PAO1 culture is bright yellow/green and opaque.

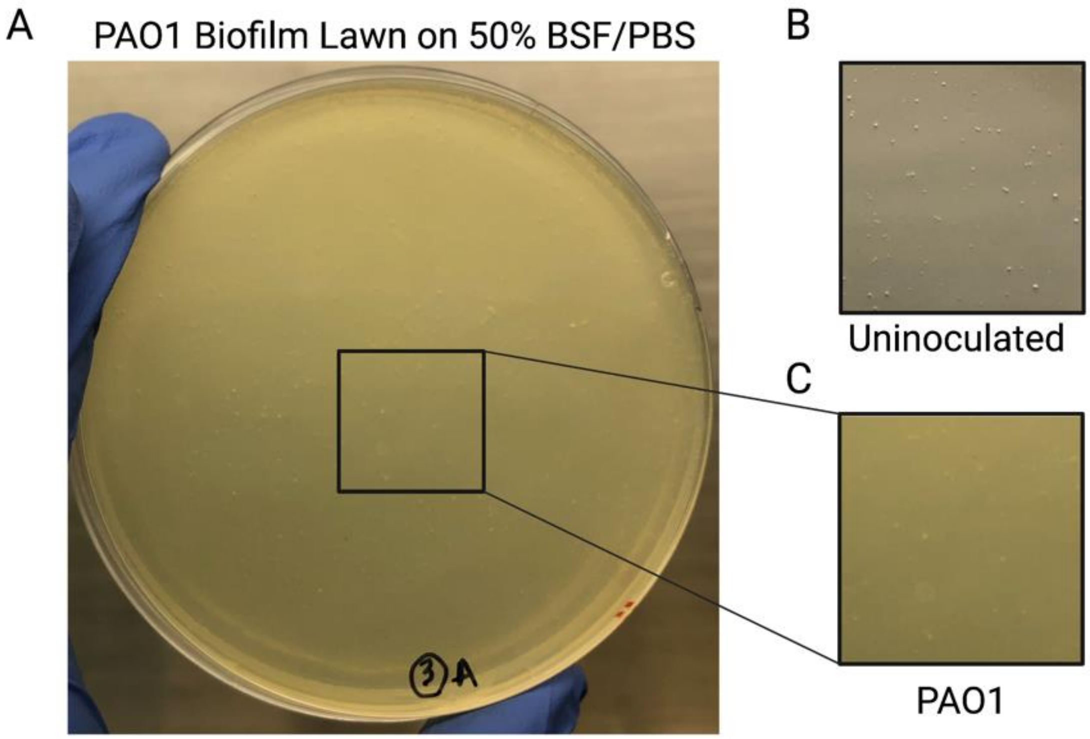

S2 Fig. Photos of the lawn biofilm grown on 50% BSF/PBS with 1.5% agar after 48 hrs of static incubation (A). Select areas of the plates without inoculated as a control (**B**) and with PAO1 (**C**) show the PAO1 biofilm has a brighter yellow/green color. The biomass generated is thinner than expected on LB agar so three plates were combined to compose one metabolomics sample.

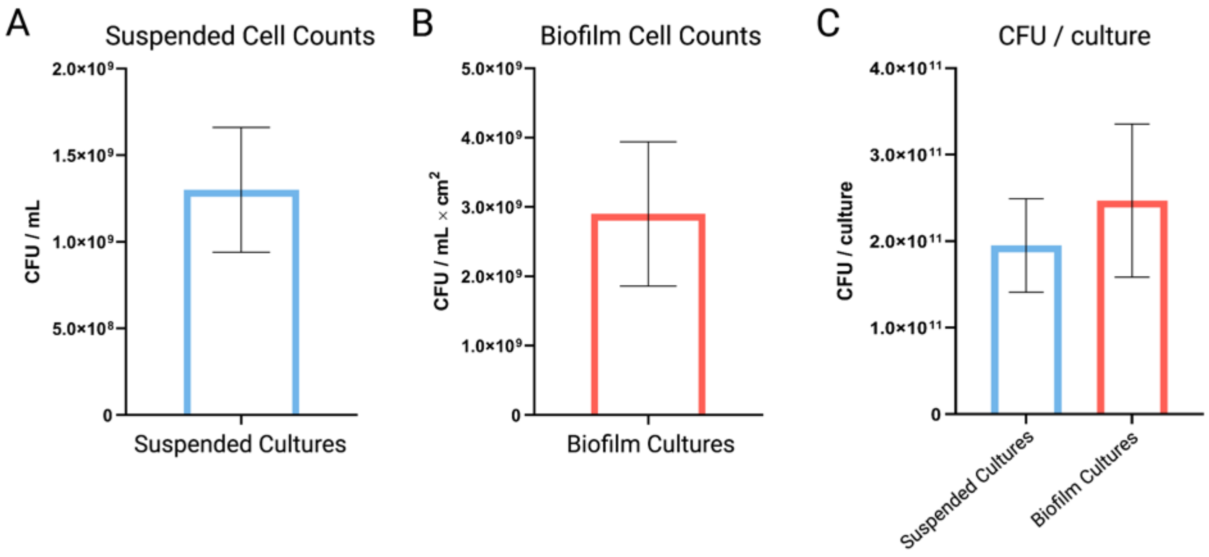

S3 Fig. Similar cell numbers as determined by CFUs were cultured from the biofilm and suspended culture samples for the untargeted metabolomics analysis. Colony-forming units (CFUs)/mL for suspended cultures (blue) (**A**) (n=3) and CFUs/mL × cm^2 for biofilm lawns (red) (**B**) (n=3) were measured by serial plate dilutions. When converted to CFU/culture (**C**) the cell count for suspended culture and biofilm was 2.5×10^{11} and 2.0×10^{11}, respectively, which was not significantly different by unpaired, two-tailed *t*-test.

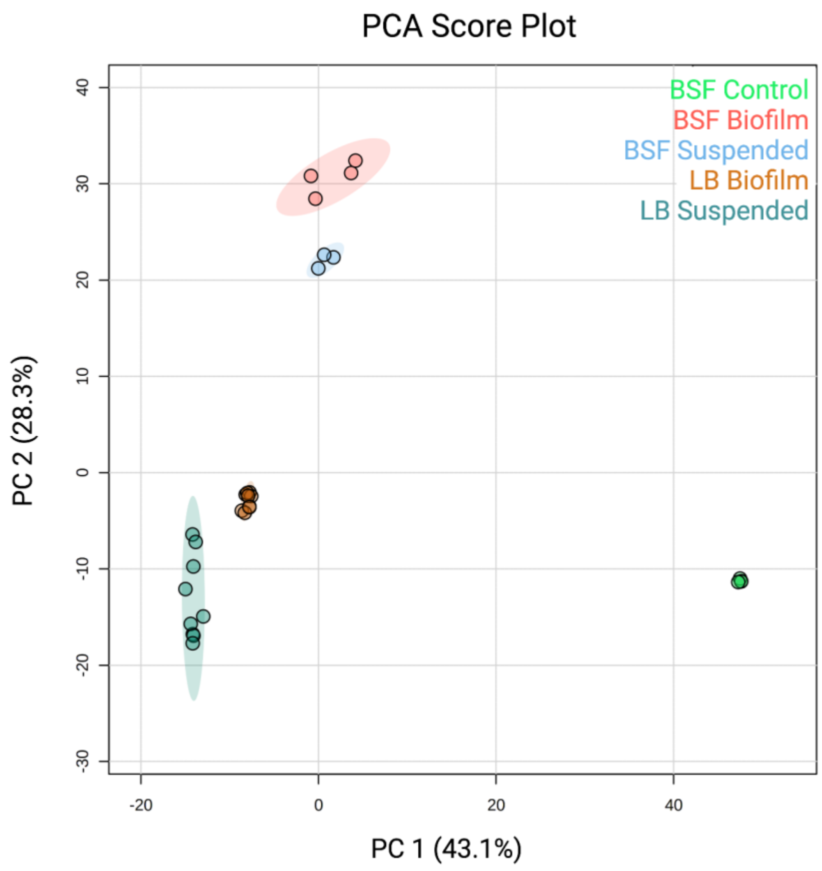

S4 Fig. The two-dimensional score plot for PCA of the uninoculated BSF controls (green) (n=4), BSF suspended cultures (blue) (n=3), BSF lawn biofilms (red) (n=4), LB suspended cultures (teal) (n=9), and LB lawn biofilms (orange) (n=9). PCA is based on the quantitation of metabolites peaks after normalization to total sum and pareto scaling showing clustering of sample cohorts with no overlap of the ellipses (ellipses represent 95% confidence intervals), displaying good separation between and repeatability within cohorts of samples. There is a clear separation between suspended culture and biofilm phenotypes regardless of media, but the separation is much greater between LB and BSF cultures.

Supplementary Tables

S1 Table. Metabolites identified and quantified from ^{13}C-^{1}H HSQC spectra of uninoculated BSF, PAO1 suspended in BSF, and lawn biofilm cultures grown in BSF.

	Control	Control	Biofilm	Biofilm	Suspended	Suspended			
**Metabolite**	**Average Metabolite Peak Volume**	**Standard Deviation**	**Average Metabolite Peak Volume**	**Standard Deviation**	**Average Metabolite Peak Volume**	**Standard Deviation**	**Fold change (Biofilm/Suspended)**	**p-value**	**FDR Test Result [a]**
Putrescine			8.29E+06	7.73E+06	2.02E+08	1.27E+07	0.04	3.12E-06	TRUE
Succinic acid			6.45E+07	1.97E+07	7.64E+08	6.35E+07	0.08	6.25E-06	TRUE
Phosphoethanolamine	4.14E+06	1.93E+06	2.60E+06	4.49E+05	2.44E+07	1.71E+07	0.11	4.08E-05	TRUE
gamma-Aminobutyric acid			1.46E+08	3.77E+07	4.77E+08	4.12E+07	0.31	9.96E-05	TRUE
Phosphorylcholine			2.60E+06	4.49E+05	3.81E+07	6.14E+06	0.07	1.22E-04	TRUE
Ethanolamine			1.86E+08	3.75E+07	4.81E+08	3.57E+07	0.39	1.76E-04	TRUE
Glycerophosphocholine			1.17E+07	3.04E+06	9.03E+07	1.51E+07	0.13	2.88E-04	TRUE
Glycerol-3-phosphate			4.44E+06	3.42E+06	6.66E+07	1.57E+07	0.07	4.13E-04	TRUE
Choline			2.60E+06	4.49E+05	3.00E+07	7.60E+06	0.09	1.15E-03	TRUE
Leucine	6.36E+07	1.28E+07	5.41E+06	3.43E+06	2.61E+07	4.12E+06	0.21	1.25E-03	TRUE
Pyruvic acid	1.58E+07	3.96E+06	1.38E+07	3.91E+06	6.17E+07	1.92E+07	0.22	1.88E-03	TRUE
D-Aspartate			2.30E+07	8.32E+06	7.47E+07	2.10E+07	0.31	1.96E-03	TRUE
alpha-Ketoglutaric acid	5.24E+07	1.07E+07	2.60E+06	4.49E+05	3.74E+07	1.26E+07	0.07	3.19E-03	TRUE
Alanine	1.05E+08	1.98E+07	2.86E+07	1.13E+07	1.83E+08	4.85E+07	0.16	3.31E-03	TRUE
L-Valine	1.25E+08	2.38E+07	9.37E+06	7.56E+06	3.61E+07	5.12E+06	0.26	4.18E-03	TRUE
beta-Alanine			2.60E+06	4.49E+05	5.24E+07	2.26E+07	0.05	4.36E-03	TRUE
1,3-Diaminopropane			2.60E+06	4.49E+05	1.31E+08	4.74E+07	0.02	4.99E-03	TRUE
Benzoate			3.70E+07	4.61E+06	1.87E+07	4.58E+06	1.98	5.00E-03	TRUE
Gluconic acid			3.39E+07	1.04E+07	6.92E+06	2.20E+07	4.90	7.08E-03	TRUE
Acetic acid	5.77E+07	8.67E+06	9.49E+07	2.45E+07	3.15E+07	1.57E+07	3.01	7.99E-03	TRUE
Lysine	1.35E+07	8.86E+06	2.60E+06	4.49E+05	1.90E+07	1.19E+07	0.14	8.45E-03	TRUE
Trehalose			1.85E+07	4.66E+06	5.82E+06	4.03E+06	3.18	9.20E-03	TRUE
L-Glutamic acid	1.89E+07	5.28E+06	2.22E+09	4.02E+08	1.28E+09	3.41E+08	1.74	1.89E-02	TRUE
L-Glutathione			9.62E+07	3.64E+07	2.73E+07	3.73E+07	3.53	2.66E-02	TRUE
Fumaric acid			6.92E+06	4.74E+06	2.16E+07	6.71E+06	0.32	2.87E-02	TRUE

Cysteine-glutathione disulfide			3.00E+07	1.67E+07	2.38E+06	1.01E+07	12.64	3.83E-02	TRUE
Creatine	1.08E+08	2.02E+07	8.52E+07	3.91E+07	2.48E+07	5.74E+06	3.43	4.80E-02	TRUE
p-Toluic acid	8.16E+06	2.66E+06	2.46E+08	4.62E+07	1.64E+08	3.43E+07	1.50	5.98E-02	False positive
AMP			2.25E+07	7.94E+06	3.51E+07	4.94E+06	0.64	6.85E-02	False positive
Creatinine	3.29E+07	6.35E+06	1.14E+07	6.59E+06	2.38E+06	2.67E+05	4.80	6.89E-02	False positive
L-Isoleucine	3.19E+07	6.57E+06	2.60E+06	4.49E+05	6.43E+06	3.54E+06	0.40	7.59E-02	False positive
Betaine	1.57E+08	2.98E+07	3.91E+08	2.40E+08	6.81E+08	6.98E+07	0.57	1.02E-01	False positive
NAD			1.01E+07	6.47E+06	1.57E+07	5.22E+06	0.64	2.64E-01	False positive
L-Threonine	4.70E+07	1.70E+07	3.41E+07	7.78E+06	2.46E+07	1.40E+07	1.39	2.69E-01	False positive
N-Acetyl-L-alanine	8.17E+06	2.63E+06	1.62E+07	2.56E+06	2.57E+07	1.51E+07	0.63	3.44E-01	False positive
Glycine	1.21E+08	2.27E+07	1.74E+08	5.88E+07	2.02E+08	4.76E+07	0.86	5.42E-01	False positive
3-Carboxypropyl trimethyl ammonium			1.28E+07	9.39E+06	1.15E+07	3.56E+06	1.11	8.34E-01	False positive
Suberic acid			1.12E+07	3.51E+06	1.07E+07	2.45E+06	1.04	8.74E-01	False positive
Mannose	1.18E+07	1.99E+06	1.56E+08	2.97E+07	1.58E+08	1.19E+07	0.99	9.19E-01	False positive
Allantoin	1.18E+07	3.13E+06							False positive
beta-gentiobiose	4.18E+08	7.84E+07							False positive
Trimethylamine-N-oxide	3.27E+06	1.77E+06							False positive
L-Tyrosine	2.02E+07	3.91E+06							False positive
L-Serine	6.46E+06	4.19E+06							False positive
L-Methionine	7.47E+06	1.61E+06							TRUE
Taurine	3.73E+06	2.36E+06							False positive
L-Phenylalanine	1.19E+07	2.57E+06							False positive
Lactic acid	1.65E+09	3.15E+08							False positive
3-Hydroxybutyrate	9.83E+07	1.90E+07							False positive

L-Glutamine	9.89E+07	1.51E+07							False positive
Glucose	6.37E+08	1.19E+08							False positive

[a] FDR = false discovery rate; TRUE = correctly rejected null hypothesis; False positive = incorrectly rejected null hypothesis
